# Building smarter surveillance: a framework for artificial intelligence-enabled monitoring of overuse injuries in youth sports

**DOI:** 10.3389/fpubh.2026.1966293

**Published:** 2026-10-07

**Authors:** Patrick Mayolo, Zachary A. Blashinsky, John Wahl, Alexander Bouterse, Felix Oettl, James A. Pruneski, Kristian Samuelsson

**Affiliations:** 1 Department of Orthopaedic Surgery, Tripler Army Medical Center, Honolulu, HI, United States; 2 Department of Orthopaedic Surgery, Balgrist University Hospital, University of Zürich, Zurich, Switzerland; 3 Sahlgrenska Sports Medicine Center, Gothenburg, Sweden; 4 Department of Orthopaedics, Institute of Clinical Sciences, Sahlgrenska Academy, University of Gothenburg, Gothenburg, Sweden; 5 Department of Orthopaedics, Sahlgrenska University Hospital, Mölndal, Sweden

**Keywords:** artificial intelligence (AI), early sport specialization, overuse injuries, sport injuries, wearable devices

## Abstract

Early sport specialization (ESS), defined as intensive, year-round training in a single sport at the exclusion of others, has become increasingly prevalent among youth athletes. This trend has coincided with a rise in overuse injuries, and premature attrition. Concurrently, advances in wearable sensors and artificial intelligence (AI) enable real-time quantification of training load, biomechanical deviations, and recovery. However, a translational gap remains: existing AI surveillance models are predominantly validated in elite adult male cohorts, leaving youth athletes underserved. To address this gap, we propose a translational four-pillar framework for ESS-aware AI injury monitoring in youth sport. Bridging technological capabilities with developmental physiology through structured AI surveillance provides a structured risk-monitoring roadmap with the potential to inform preventative strategies, optimize physical readiness, and support long-term musculoskeletal health in youth athletes.

## Introduction

Early sport specialization (ESS) has become increasingly prevalent among youth athletes over the past decade (1, 2). This trend has coincided with a parallel rise in overuse injuries as repetitive unidirectional loading, training monotony, and insufficient recovery accumulate musculoskeletal damage in the developing athlete (3). The consequences extend beyond acute injury to include psychological burnout and premature attrition from sport (3). The public health stakes are considerable, as overuse injury drives healthcare utilization, threatens sustained physical activity participation, and may compromise long-term musculoskeletal health into adulthood (4).

In parallel with these epidemiological trends, the proliferation of wearable sensors and artificial intelligence (AI) has generated considerable enthusiasm for continuous, data-driven injury surveillance in sport (5, 6). Inertial measurement units, heart rate variability monitors, and machine learning classifiers now permit quantification of training load, recovery, and biomechanical deviation with a granularity previously unattainable (7, 8). However, this abundance of data has not translated into clinically meaningful outcomes, and a persistent gap remains between technological capability and demonstrable patient benefit (7). Compounding this translation gap, the overwhelming majority of these tools have been developed and validated in elite, adult, and predominantly male athletes (5, 9). As such, youth athletes remain an underserved population in AI sports research, despite bearing a substantial share of the overuse injury burden.

Addressing this gap requires more than incremental technical refinement. To date, no framework integrates the developmental biology of ESS, the capabilities and limitations of AI-based monitoring, and the governance structures necessary to deploy such tools responsibly in youth populations. The result is a persistent disconnect among the stakeholders who must ultimately collaborate, including technology developers, sports medicine clinicians, coaches, and policymakers, each of whom operates with a distinct vocabulary and set of priorities. In this article, we propose a translational, equity-conscious framework for ESS-aware AI injury monitoring in youth sport, intended to align these constituencies around a common set of principles and to ensure that the benefits of AI-driven surveillance are realized without widening existing disparities in youth athlete health. The novelty of this framework lies in its integration of developmental physiology, ESS-specific workload patterns, AI-tractable biometric and biomechanical signatures, population-specific validation requirements, and governance considerations for minors into a single, unified model tailored specifically to youth athletes. This framework is intended as a risk-stratification and monitoring model, not a validated injury-prevention tool.

## Methods

This manuscript is a narrative review intended to synthesize current evidence on wearable-sensor and AI-enabled monitoring of overuse injuries in youth athletes and to propose a conceptual framework tailored to early sport specialization. Literature was identified through iterative searches of PubMed using combinations of terms including “youth sports,” “early sport specialization,” “overuse injury,” “wearable sensors,” “machine learning,” and “injury prediction.” Searches focused on publications from 2000 to 2026, with additional studies identified through reference lists and targeted follow-up searches. Included studies were selected based on relevance to ESS physiology, wearable-sensor modalities, AI-based injury-risk modeling, or governance considerations for minors.

### ESS and the overuse injury phenotype

ESS is often defined using a 3-point classification scale originally developed by Jayanthi et al. (10). The scale classifies athletes as low, moderate, or high specialization based on three criteria: year-round training (>8 months/year in a single sport), choosing a main sport, and refraining from other sports to focus on one (10). Each criterion scores one point, with a score of 3 correlating with highly specialized. This definition has subsequently been recognized and adopted by multiple organizations to include the American College of Sports Medicine (ACSM) and American Medical Society for Sports Medicine (AMSSM) (11, 12).

ESS is distinct from other sport specialization patterns such as early diversification or late specialization. In early diversification, or, sport sampling, the athlete participates in multiple sports during childhood at lower intensity followed by gradual narrowing to a primary sport around age 12–13 years (11, 13). In contrast, late specialization typically occurs after age 14–15 years. ESS prevalence varies by sport and is more common in individual sports compared to team sports, and it is estimated that between 10 and 30% of youth athletes in the United States specialize in a single sport (11). Gymnastics, tennis, swimming/diving, and soccer athletes are more likely to specialize early, while football and baseball athletes are more likely to specialize late (14). Additionally, athletes may meet the criteria for early sports specialization but not self-identify as such (1).

Overuse injury in specialized youth athletes is caused by cumulative microtrauma from repetitive loading without sufficient recovery (15). The immature skeleton is less tolerant of stress and the period around peak height velocity (PHV) is considered the highest-risk window for overuse injuries. Rapidly growing physes, apophyses, and articular surfaces are more sensitive to tensile, shear, and compressive forces (3, 16). Sport-specific examples of overuse injuries include “gymnast’s wrist,” describing distal radius epiphysiolysis from repeated axial loading and dorsiflexion, and “little league shoulder,” describing proximal humeral epiphysiolysis from excessive torsional and distraction forces in baseball (3). A recent systematic review of primary periphyseal stress injuries found that cross-sectional prevalence was high, 10–83% in gymnasts’ wrists, 0–36.6% in baseball shoulders, and 52.6% in platform divers’ wrists, and growth disturbance was recorded in 12.7% of athletes with primary periphyseal stress injuries (17). This absence of cross-training stimuli can cause asymmetric musculoskeletal adaptation; Palaiothodorou et al. demonstrated that bone mineral content and bone area were asymmetric in the dominant arm of youth tennis players, and that training time was the strongest predictor of bone asymmetry (18). Other common sport-specific overuse injuries include medial elbow injuries in baseball/throwers (epicondyle apophysitis, avulsion fractures, ulnar collateral ligament pathology), spondylolysis and capitellar osteochondritis dissecans in gymnasts, and stress fractures in runners.

Current guidelines from the ACSM recommend adequate rest from sport to prevent overuse injury, including 2–4 months off annually and a sports training-to-free-play ratio of less than 2:1 h per week (12). Decreased sleep volume, increased training intensity, and increased training load produce a dose–response increase in injury risk, and athletes with all three risk factors have a significantly higher risk of sustaining an overuse (19). Among elite youth athletes, those consistently obtaining more than 8 h of sleep on weekdays reduced their odds of injury by 61%, and poor sleep quality has been identified as the single strongest predictor of new injury in collegiate distance runners (20). Additionally, training volume thresholds can be calibrated to the athlete’s developmental stage, and the ACSM recommends that weekly training hours should not exceed the athlete’s age in years (12). Most of these physiologic signatures of early sports specialization and associated overuse injuries are quantifiable, longitudinal, and multimodal, making them amenable to monitoring and regulation by AI-driven surveillance systems (Figure 1).

**Figure 1:**
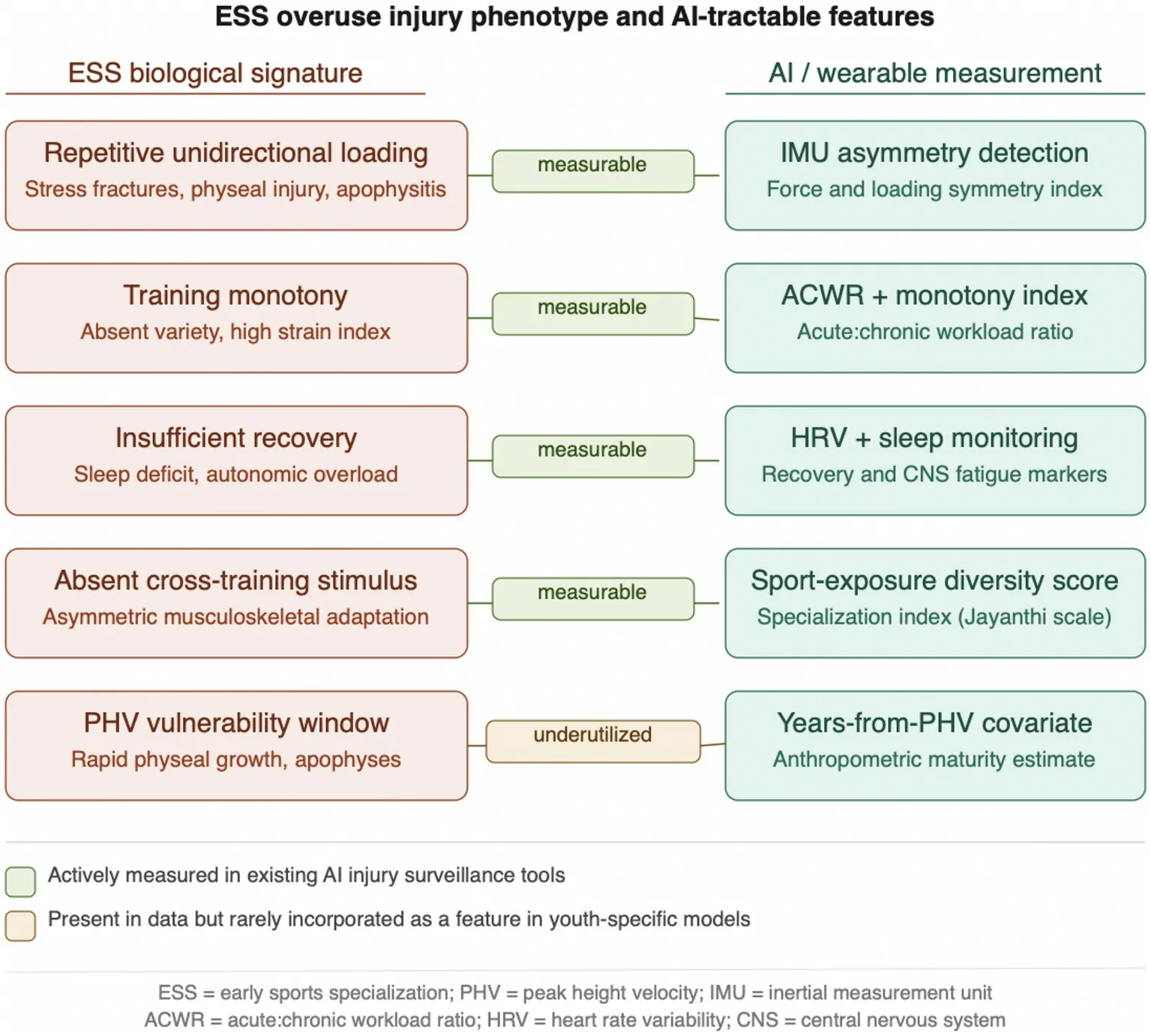
ESS overuse injury phenotype and corresponding AI-tractable features. Five core biological signatures of early sports specialization (ESS) associated with overuse injury risk are mapped to their corresponding artificial intelligence (AI) and wearable sensor measurement targets.

### Current AI and wearable monitoring in youth athletes

Wearable sensors are small electronic and mobile devices with wireless communication or data storage capabilities that can be used to collect performance data (21). These are often combined with AI learning algorithms to quantify and guide aspects of performance, including training, injury prevention, and recovery.

#### Current wearable technologies and sensor modalities

Categories of available sensors and modalities include motion sensors, biometric, bioelectric, optical, and chemical sensors, and environmental and mechanical sensors (22). Of motion sensors, the most common data measured is acceleration (via accelerometers) and angular acceleration (via gyroscopes). When accelerometer and gyroscope data are combined, Inertial Measurement Units (IMUs) are generated, which can simultaneously measure displacement, velocity, and angular acceleration to quantify movements such as gait characteristics, jump-landing mechanics, and impact forces (7, 22). These built-in sensors can be combined with wearable Global Positioning System (GPS) technology to provide more advanced measurements (7). Biometric sensors include non-invasive lactate, oxygen, and hydration sensors (7, 22). There is also emerging technology for continuous non-invasive blood glucose monitoring, however there are no FDA-approved devices to date.

Bioelectric sensors measure signals from electrical activity, sound waves, or respiratory activity. The most common are sensors that capture electrocardiographic activity to measure heart rate, heart rate variability, and assess arrhythmias. This can indirectly measure workload, recovery, overall fitness status, and central nervous system fatigue (7, 22). Respiratory sensors include bands that measure respiratory rate and indirectly measure tidal volume, as well as wearable masks that evaluate end-tidal CO2, providing data on workload, recovery, and aerobic versus anaerobic utilization during activity. Electromyography (EMG) sensors specifically evaluate muscular activation and utilization. These wearable sensors assess overall muscle activation and patterns of muscular activity during specific exercises, and screen for muscular fatigue and imbalance by detecting decreased or asymmetric activation in response to a stimulus (7, 22). Electroencephalography (EEG) sensors measure neural signals to assess attention and thought-process patterning in response to challenges or specific sport-specific exercises. Often combined with EEG measurements are electrooculography (EOG) sensors that assess gaze direction to evaluate for focus and search patterns during sport-specific exercises (22). Acoustic sensors are also occasionally used in team sports to monitor trends in communication. Finally, in the bioelectric category are electrodermal activity (EDA) sensors that utilize galvanic skin response sensors to measure skin temperature and assess increased activity and exhaustion that may indicate an impending injury in an athlete.

Environmental and mechanical sensors include piezoelectric sensors, strain gauges, and capacitive sensors for evaluating force and pressure. These measurements are used to calculate strike force, weight-bearing areas, and strength asymmetries (7). Newer sensor technologies include flexible and textile sensors that detect subtle motion in real time for use in training coordinated and precise movements (7).

There are many commercially available products on the market utilized by both elite and recreational athletes. Accordingly, there have been a growing number of publications evaluating wearable data and injury risk in adult cohorts. Skazalski et al. used a commercially available wearable device to monitor jump counts, heights, and landing forces in adult volleyball players to anticipate impending overuse injuries (23). Wearable technologies have been used to assess wrist position and movement in golfers, critically evaluating swing form (24). Numerous studies have evaluated the use of motion and force sensors to assess running biomechanics and training optimization in track and cross-country athletes (25–27). Finally, wearable sensors have been used to assess and optimize swimmers’ velocity and water displacement to improve stroke mechanics (28). Sensors also have a role during athletic events, and were used to optimize athlete performance between events during the 2020 Tokyo Olympic Games (29). After an event, sensors can detect athletes’ fatigue, recovery status, sleep, and physiologic reserves, allowing trainers and coaches to evaluate readiness to return to full training (5).

While there is a growing body of literature supporting the use of wearable sensors in adult athletes, there remains limited literature focusing on pediatric and adolescent athletes. Most published works in these cohorts evaluate the use of wearable sensors to screen for and detect brain injuries in contact sports, as well as on overall outcomes and recovery. Brennan et al. used accelerometers implanted into helmets of high school athletes and found that increased mean peak linear head acceleration and rotational head acceleration were linked with concussion events (30). Ransom et al. used wearable sensors to evaluate sleep quality and daily activity in adolescent athletes in the weeks following a concussion and found more light sleep, more nocturnal wake time, and lower daily activity (31). Wearable devices have also been described for use in evaluating running mechanics and the physiologic response to training in adolescent athletes (32–34).

Despite the growing body of literature evaluating the use of wearable sensors, there are still limitations that warrant continued investigation. Primarily, there is a gap in delivering information and recommendations to athletes in real time, with much of the data only available for review after training sessions or competitions. Additionally, there are inconsistencies in the reliability and accuracy of the collected data across devices; in head-to-head comparisons against electrocardiographic reference standards, no wrist-worn optical heart rate monitor achieved the accuracy of a chest strap, with concordance correlation coefficients ranging from 0.996 for a chest-strap monitor down to 0.67 for certain wrist devices, and accuracy degrading systematically as exercise intensity increased (35). Similarly, consumer sleep trackers demonstrate mean absolute percentage errors of 8.1–16.9% for total sleep time and remain insufficient for reliable sleep staging, with polysomnography retained as the gold standard (36). Finally, there remains a paucity of validated studies on their use, eliciting the need for larger-scale studies to validate the efficacy and utility of wearable sensors and AI devices in adolescent athletes.

### A four-pillar framework for AI-assisted ESS overuse injury detection

To address this gap, we propose a translational four-pillar framework for ESS-aware AI injury monitoring in youth sports (Figure 2):

**Figure 2:**
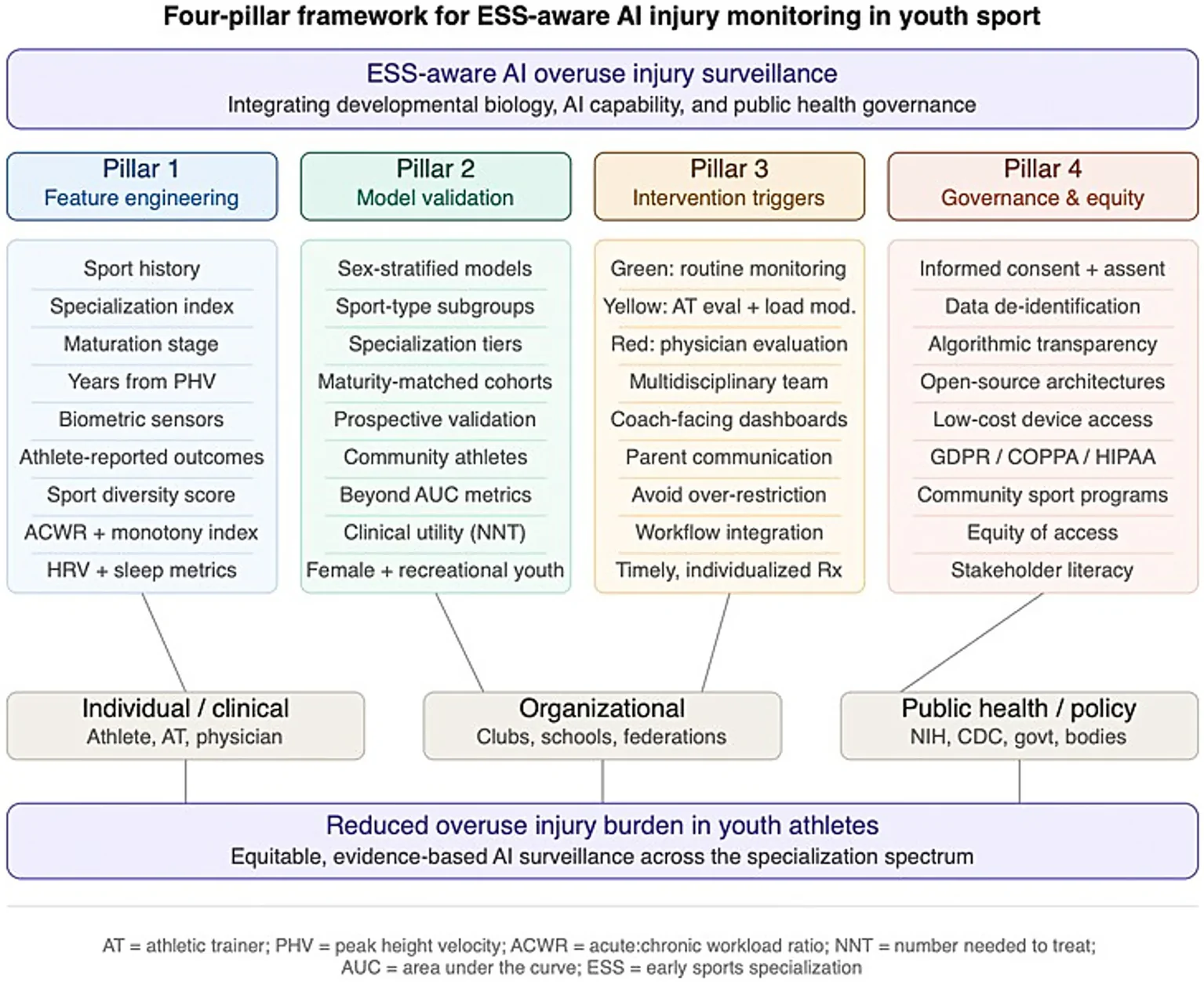
Four-pillar framework for ESS-aware AI injury monitoring in youth sport. The framework organizes ESS-aware AI overuse injury surveillance around four operational pillars: (1) ESS-aware feature engineering; (2) population-specific model validation; (3) tiered intervention triggers; and (4) governance, privacy, and equity provisions. The four pillars interface with three implementation levels converging on the shared goal of reducing overuse injury across youth athletes. Unlike prior AI or wearable‑based injury‑surveillance approaches, which typically address these elements independently, this framework integrates them across individual, organizational, and public‑health levels to create a coordinated, youth‑specific structure.

#### Pillar one: ESS-aware feature engineering

The optimal detection model for overuse injuries is one that provides both a robust, dynamic assessment of the biomechanical and physiologic stresses exerted on the developing musculoskeletal system while also accounting for individual work volumes and physiologic maturity. We propose the following core feature domains as necessary inclusions within the structure of such a model:

##### Sports history and specialization

As risk for overuse injuries increases commensurately with increasing sports specialization, young athletes’ degree of ESS should be appropriately measured and stratified within detection algorithms. The 3-point classification system proposed by Jayanathi and colleagues presents the most objective quantification of this variable to date and serves as an ideal inclusion for determining athletes’ level of specialization and their subsequent risk profile (10).

##### Growth and maturation stage

Skeletal maturity and peak growth velocity have been proposed as an objective measure of physiologic maturation and may serve as an optimal screening tool for stratifying athletes at risk for overuse injuries within predictive models. Specifically, years from peak height velocity, estimated through predictive equations, was identified as the most influential predictor of overuse injuries among elite youth soccer players in a recent machine learning analysis by Rommers et al. (8, 37).

##### Biometric data and athlete-reported outcomes

A combination of wearable sensor-obtained objective data and athlete-reported functional outcomes are essential components of risk-stratification models, as they capture dynamic changes in physiologic and functional status throughout in-season training workloads. A particularly well-validated instrument for capturing athlete-reported outcomes in this context is the Oslo Sports Trauma Research Centre Overuse Injury Questionnaire (OSTRC-O), which was able to identify substantially more overuse problems than conventional injury registration methods (38). Nordic cohorts employing the OSTRC-O have achieved response rates exceeding 95–97% (39, 40), confirming its feasibility as a longitudinal surveillance tool in youth sport settings. Importantly, this infrastructure represents a ready-made, high-quality data source for the population-specific validation pipelines described in Pillar 2, bridging the feature engineering and validation pillars in practice.

Beyond selecting which signals to capture, the manner in which workload is modeled materially affects predictive performance. The acute-to-chronic workload ratio is conventionally partitioned into a relative low-risk range of approximately 0.8–1.3 and an elevated-risk range above 1.5, and exponentially weighted moving average formulations demonstrate greater sensitivity for detecting increases in injury likelihood than simple rolling averages, as the latter assume all workload within a period is equivalent and fail to account for decay in fitness (41). However, these ranges should not be interpreted as universally validated thresholds, as ACWR behavior varies substantially across sports, age groups, sexes, maturation stages, and workload-calculation methods, and its predictive value remains context-dependent. For ESS populations in particular, joint-specific rather than whole-body load accounting warrants consideration, as sleeve-mounted sensors capable of quantifying elbow valgus torque in throwing athletes have shown close agreement with laboratory motion capture and permit acute and chronic workload to be computed for the specific anatomic structure at risk (41). Given that overuse pathology in specialized youth athletes is anatomically localized to the repetitively loaded segment, whole-body surrogates such as total distance or global player load may systematically under-represent the tissue-level stress that drives injury in this population.

#### Pillar two: Population-specific model validation

While a number of AI-supported prediction models have been developed for prediction of sports-related overuse injuries, the vast majority of these tools are targeted toward adult athletes, with validation performed amongst elite, adult male athletes, thereby limiting their applicability to the unique biomechanical and development demands of younger and more physiologically vulnerable populations (42). A recent systematic review by Mora et al. evaluating such models found that broad generalizability was inherently limited by inclusion of heterogeneous populations and nonuniform outcome reporting, thereby constraining their extensibility to the distinct factors at play in youth athletes (43). Dedicated, prospective validation of proposed models among ESS athletes must be undertaken to establish their accuracy, generalizability, and clinical utility within this high-risk population.

This may take the form of separate validation pipelines in order to provide stratification based on degree of sports specialization, sex, level of competition, and stages of skeletal maturation. The machine learning analysis performed by Rommers et al. provides a framework for this concept by assessing injury risk in elite male soccer players aged 10–15 years, yet falls short of addressing risk stratification for distinct populations of female athletes and those of varying competition tiers (8). Juahainen et al. prospectively evaluated machine learning models in a mixed-sex cohort of 314 young basketball players, however their analysis was limited by inherent difficulty with model performance even when consistent risk factors for injury were identified (44). Perhaps, the most meaningful endeavor into this field is represented by the Sport Training Assessment of Risk (STAR) tool proposed by Schley and colleagues, which specifically examined youth and collegiate athletes according to sports specialization, sex, physiologic maturity, and individualized workloads; however this, too, was limited by marginal predictive efficacy, again underscoring the need for more robust, population-specific model development and prospective validation (45).

To strengthen future ESS-focused injury-risk models, validation should extend beyond global accuracy. Robust evaluation requires assessment of discrimination, calibration, sensitivity and specificity, precision and positive predictive value, and explicit handling of class imbalance, given the low incidence of many youth overuse injuries. Performance estimates should include confidence intervals, and models should undergo external validation across sex, specialization level, competition tier, and maturation stage. Finally, decision-curve analysis or other clinical-utility frameworks should be incorporated to determine whether model outputs meaningfully improve decision-making compared with existing practice.

A recent cautionary example from Europe further underscores these limitations. Haller et al. deployed a support vector machine trained on GPS load metrics, blood biomarkers, wellness questionnaires, and physical performance tests in 25 elite under-18 soccer players, achieving injury prediction precision of only 11.1% and illness prediction precision of 4.8% (46). The authors conceded the approach was likely not cost effective or practical for non-elite clubs, precisely the population bearing the greatest ESS-related overuse burden. This study exemplifies the validation failures Pillar 2 seeks to address: small, homogeneous, elite-only training samples that produce models with negligible generalizability to the average youth athlete. Accordingly, future work should aim to explore these gaps in risk modeling by developing and prospectively validating models that account for differences in injury risk across the unique profile of contributing factors inherent to youth athletes.

#### Pillar three: Tiered intervention triggers

From the insights produced by predictive injury models, conceptual tiers of athlete-specific interventions may be considered for at-risk individuals. Specifically, stratification of low-, medium-, and high-risk athletes according to their predicted risk of impending overuse injury can guide the implementation of tailored preventive measures, ranging from routine biometric monitoring to targeted workload modification and clinical evaluation. One such risk-stratification framework has previously been posited by Qian and colleagues, who utilized random forest algorithms to group athletes into “green,” “yellow,” and “red” risk categories based routine physiological assessments, biometric markers, and current training volume (47). Notably, their model identified 85% of high-risk athletes, on average 1 week before their performance decline, suggesting that timely responses may help identify elevated risk earlier, though prospective validation is still required.

Empirically derived workload thresholds can provide initial anchors for such tiers. In elite youth male soccer players monitored across two seasons with GPS and accelerometry, non-contact injury risk was greatest when a very high number of accelerations accumulated over 3 weeks, and risk was also elevated when substantial high-speed distance was accrued against a low chronic high-speed training base (48). Thresholds of this kind offer a starting point for calibrating tier boundaries, though they require re-derivation within youth cohorts stratified by specialization status, sex, and maturity. Equally important, tiered triggers should never be driven by a single variable. Accordingly, escalation within our framework should require concordance across multiple domains, such as load, recovery, athlete-reported symptoms, and maturation status, rather than breach of any single index.

Within these frameworks, relevant stakeholders, including athletes, parents, coaches, athletic trainers, and team physicians, can be informed of an athlete’s risk of impending overuse injury and implement timely, individualized risk-mitigation strategies. For instance, athletes flagged as “moderate-risk” could undergo a comprehensive evaluation by assigned athletic trainers to identify areas of overuse, while also adjusting training loads and partnering with parents to ensure adherence to recommended recovery practices. Meanwhile, athletes deemed “high-risk” within injury prediction models, could, in contrast, be directly evaluated by a sports medicine physician, allowing for mandatory reductions in training volume, implementation of immobilization, or formal physiotherapy evaluations, if indicated. In this manner, through incorporating individualized estimates of injury risk with a tiered, multidisciplinary treatment framework, teams may use risk-stratification outputs to guide monitoring intensity, while avoiding unnecessary restriction among athletes with lower risk profiles. These examples are intended as hypothetical applications of risk-stratification outputs; any clinical or training decisions linked to model-generated risk tiers would require rigorous prospective validation to assess false-positive and false-negative consequences.

#### Pillar four: Governance, privacy, and equity

Though AI-driven injury prediction models carry the potential to substantially lessen the physiologic burden imposed on young athletes, their integration into both recreational and competitive sport environments requires careful oversight, particularly among resource-limited populations. Efforts to promote equitable access should include open-source model architectures and equitable device provision for under-resourced schools and community programs, as well as compatibility with consumer-grade wearable devices rather than requiring specialized hardware.

Additionally, enhanced monitoring of youth athletes presents the potential for undue breaches in privacy sensitive biometric data being collected and shared without adequate safeguards or informed consent. As such, participants should be adequately counseled on the nature, purpose, and potential downstream uses of their data, and appropriate protections should be implemented in order to preserve confidentiality and subject autonomy throughout the data-collection, storage, and analysis process.

Importantly, the regulatory landscape for AI tools in youth sport remains incompletely defined. A private club’s injury-risk monitoring system may not meet the threshold for high-risk classification under regulatory guidelines, such as the European Union (EU) AI Act, which targets employment, education, and essential services. This leaves risk monitoring systems in a regulatory gray zone under that framework. However, national provisions governing biometric and health data, as well as heightened protections for minors, apply regardless of AI Act classification and impose meaningful obligations around consent, data minimization, and purpose limitation. Manuscripts and implementation efforts in this space should explicitly acknowledge this ambiguity rather than assuming high-risk classification, and should engage legal and ethics expertise early in system design to navigate the gap between what the law currently requires and what responsible deployment demands.

In addition to these governance principles, AI systems deployed in youth sport must incorporate safeguards addressing algorithmic bias across sex, age, maturation stage, and socioeconomic groups; routine fairness assessment; and monitoring for data drift as athletes grow and training environments evolve. Clear accountability structures should define responsibility for misclassification, particularly when false-positive or false-negative outputs influence training or clinical decisions. Finally, human-in-the-loop decision-making should remain central to any AI-supported workflow, ensuring that coaches, athletic trainers, and clinicians interpret model outputs within the broader context of athlete development and clinical judgment.

### Implementation pathway and future directions

Implementing this model in youth sports requires a unified and accessible data infrastructure. This should include specialization history, maturation stage, wearable-derived data, and athlete reported outcomes. Ideally, these data should be accessible across sports organizations, to include community, club, and scholastic sponsored organizations. This will allow for longitudinal monitoring without excessive administrative burden. Once created, multi-site validation that stratifies risk across sex, sport type, competition level, and maturity stage will be needed to improve generalizability and reduce bias.

Effective implementation also depends on stakeholder literacy and robust governance. Coaches, parents, athletes, and administrators must be trained to interpret risk, understand the rationale behind workload adjustments, and appreciate the importance of accurate self-reporting. Clear policies governing data ownership, privacy, and consent, particularly for minors, are essential to maintaining trust and preventing misuse of biometric information. Equitable access must remain a priority, with open-source model architectures, low-cost device options, and partnerships that support under-resourced schools and community programs. In the United States, there is significant disparity of sports medicine resources for youth sports by socioeconomic status (49, 50). Accordingly, affordable AI-based monitoring systems can help improve access to diagnostic healthcare for athletes across all socioeconomic levels.

## Conclusion

The signatures of ESS, including loading asymmetry, training monotony, recovery deficits, and diminished sport-exposure diversity, are inherently measurable and therefore amenable to AI-based detection. Current tools, however, remain poorly calibrated for youth cohorts. The four-pillar framework proposed in the current study, encompassing ESS-aware feature engineering, population-specific validation, tiered intervention triggers, and robust governance, offers a structured risk-monitoring roadmap that may help guide future efforts to close this gap. Realizing its potential will require coordinated efforts from researchers, clinicians, developers, and policymakers alike. Ultimately, reducing the burden of overuse injury in youth athletes is not merely a technical challenge but a population health imperative, and building smarter, more equitable surveillance is a necessary step toward meeting it.
